# Integrative analysis of survival-associated gene sets in breast cancer

**DOI:** 10.1186/s12920-015-0086-0

**Published:** 2015-03-12

**Authors:** Frederick S Varn, Matthew H Ung, Shao Ke Lou, Chao Cheng

**Affiliations:** Department of Genetics, Geisel School of Medicine at Dartmouth, Hanover, New Hampshire 03755 USA; Institute for Quantitative Biomedical Sciences, Geisel School of Medicine at Dartmouth, Lebanon, New Hampshire 03766 USA; Norris Cotton Cancer Center, Geisel School of Medicine at Dartmouth, Lebanon, New Hampshire 03766 USA

**Keywords:** Breast cancer, Gene sets, Prognosis, Survival prediction

## Abstract

**Background:**

Patient gene expression information has recently become a clinical feature used to evaluate breast cancer prognosis. The emergence of prognostic gene sets that take advantage of these data has led to a rich library of information that can be used to characterize the molecular nature of a patient’s cancer. Identifying robust gene sets that are consistently predictive of a patient’s clinical outcome has become one of the main challenges in the field.

**Methods:**

We inputted our previously established BASE algorithm with patient gene expression data and gene sets from MSigDB to develop the gene set activity score (GSAS), a metric that quantitatively assesses a gene set’s activity level in a given patient. We utilized this metric, along with patient time-to-event data, to perform survival analyses to identify the gene sets that were significantly correlated with patient survival. We then performed cross-dataset analyses to identify robust prognostic gene sets and to classify patients by metastasis status. Additionally, we created a gene set network based on component gene overlap to explore the relationship between gene sets derived from MSigDB. We developed a novel gene set based on this network’s topology and applied the GSAS metric to characterize its role in patient survival.

**Results:**

Using the GSAS metric, we identified 120 gene sets that were significantly associated with patient survival in all datasets tested. The gene overlap network analysis yielded a novel gene set enriched in genes shared by the robustly predictive gene sets. This gene set was highly correlated to patient survival when used alone. Most interestingly, removal of the genes in this gene set from the gene pool on MSigDB resulted in a large reduction in the number of predictive gene sets, suggesting a prominent role for these genes in breast cancer progression.

**Conclusions:**

The GSAS metric provided a useful medium by which we systematically investigated how gene sets from MSigDB relate to breast cancer patient survival. We used this metric to identify predictive gene sets and to construct a novel gene set containing genes heavily involved in cancer progression.

**Electronic supplementary material:**

The online version of this article (doi:10.1186/s12920-015-0086-0) contains supplementary material, which is available to authorized users.

## Background

Accurate cancer prognosis offers patients an approximation of the prospective clinical outcome of their disease while aiding physicians in developing treatment plans. Traditionally, prognoses have been based on histological features of patient tumors [1]. However, these histology-based predictions fail to account for the genetic heterogeneity between cancer samples, leading to varied prediction accuracy. Understanding the unique molecular profile of each patient’s cancer may improve cancer prognosis accuracy and further the development of personalized cancer therapies [1]. To this end, several genetic biomarkers have been defined for use in the clinic as indicators of cancer prognosis [1]. Initially, these markers were single genes whose expression was correlated with clinical outcome [1]. There now exist several multi-gene signatures that have been developed to improve upon the predictions of single-gene markers [2-16]. Many of the gene sets are composed of individual genes whose expression is correlated with disease outcome, while others are based on knowledge of fundamental cancer mechanisms that are believed to play a role in patient survival time. In breast cancer, predictive signatures have been developed extensively. Some of these signatures, such as NKI70 [2], Oncotype DX [3], and PAM50 [17], have been approved for use in the clinic. By combining the expression of these genes with information on each patient, such as subtype of cancer and treatment status, a more accurate prognosis can be made [18]. We argue that it will be useful to systematically investigate the effectiveness of these gene sets in predicting breast cancer prognosis.

Previous studies have integrated signatures to examine their combined effect on prognosis accuracy [19,20]. These studies show that the integration of different prognostic signatures can significantly improve the accuracy of clinical outcome prediction. However, these studies focused on a small number of well-characterized gene signatures in breast cancer. Expanding this analysis to a larger number of signatures can provide greater insight into the molecular basis for a patient’s cancer. One resource that can aid in this analysis is MSigDB [21]. MSigDB is an online database of gene sets that have been curated across many different genome-wide studies. These gene sets capture genetic regulation across different biological contexts, many of them relating to the hallmarks of cancer such as cell proliferation, apoptosis, and DNA repair [22]. Many previously reported prognostic signatures are included in this database, such as the 70-gene signature by Van’t Veer et al. [2] and the 76-gene signature by Wang et al. [8] By combining these signatures and biological pathways in one place, MSigDB provides a resource that allows us to systematically investigate thousands of gene sets for their prognostic significance by applying them to patient gene expression datasets that include survival information.

We have previously developed an algorithm called BASE (binding association with sorted expression) to infer the regulatory activity of a transcription factor (TF) based on the expression levels of its target genes [23,24]. Using this method, we have successfully defined an E2F4 target gene-based signature for predicting survival times of patients with breast cancer [25]. We have shown that BASE can accurately infer E2F4 activity in cancer samples based on the target gene set, which stratifies breast cancer patients into good and poor prognosis groups. Similar to GSEA analysis, this method can be modified and extended to calculate a score for any gene set. The score, which we have termed the gene set activity score (GSAS), is a non-linear summary of a gene set’s component gene expression levels. This metric is a useful medium by which we can stratify patients for survival analysis, which allows us to distinguish gene sets that play a role in prognosis from those that do not.

Here, we apply the BASE algorithm to systematically investigate the association of 4,257 gene sets from MSigDB with patient survival. We begin by demonstrating applications of this technique that can be used to compare gene sets across datasets. In the first application, we use our analysis to identify and characterize 120 robustly predictive gene sets that remain significantly correlated with patient survival time across each dataset tested. In the second application, we use the GSASs to select features in one dataset that we use to classify metastasis status in multiple validation datasets, achieving high prediction accuracy. In the second part of our study, we explore the shared gene relationship between survival-associated gene sets on MSigDB. We create a gene set network based on genetic overlap between the gene sets of MSigDB and develop a novel proliferation-based gene set based on the network topology. We find that the GSASs for this gene set are significantly correlated with patient survival across all datasets tested. Furthermore, classification using the proliferation-based gene set’s component genes as features achieves a high accuracy score in predicting metastasis status. Finally, we remove the proliferation-based gene set’s genes from all the gene sets on MSigDB and find that a gene set’s predictive ability is related to the number of genes it has from the proliferative-based gene set.

## Methods

### Dataset and data processing

Breast cancer gene expression datasets were downloaded from ROCK Breast Cancer Functional Genomics [26]. Each dataset chosen contained at least 150 samples and included standard clinical data and survival outcome information (either overall survival “os”, distant metastasis free survival “dmfs”, or relapse free survival “rfs”). Five datasets [8,10,27-29] and two sub-datasets derived from a meta-analysis study [26] were downloaded in all, resulting in a collection of 1,591 unique samples across seven independent datasets. Either one- or two-channel arrays were used to measure gene expression of samples in each dataset.

Gene set data was collected from MSigDB (http://www.broadinstitute.org/gsea/msigdb) [21]. All gene sets downloaded were from the C2 curated gene sets collection, which contains 4,257 gene sets collected from pathway databases, literature, and the knowledge of domain experts. The gene set data was stored in a binary matrix with rows representing genes and columns representing gene sets. A dummy variable was used to indicate presence or absence of a gene in a gene set.

### Calculation of the activity score of gene sets

For each dataset, patient gene expression data and the list of gene sets downloaded from MSigDB were inputted into the BASE algorithm [23,24]. Briefly, the BASE algorithm sorts genes by their relative expression levels in a given sample and generates two cumulative distribution functions to encapsulate the expression activity of a target gene set (foreground function) and a complementary gene set (background function). BASE then calculates the maximum division of the two distribution functions to get a preliminary score. This preliminary score is similar in concept to the D-statistic of the Kolmogorov-Smirnov test and is representative of the expression activity of the target gene set in a given sample, with a higher preliminary score indicating higher gene set activity and a lower preliminary score indicating lower gene set activity. BASE then normalizes the preliminary score against the average of the absolute value of the preliminary scores from 1,000 permuted gene sets of equal size to the target gene set. The resulting metric is referred to as the gene set activity score (GSAS). For each sample in a given dataset, this analysis was repeated for every gene set from the C2 curated gene set collection of MSigDB.

### Survival analysis

We measured the correlation between gene set activity and patient survival outcomes using Cox proportional hazards models. A univariate model was fitted to examine the relationship between GSAS and survival time, and an additional multivariate model was fitted to account for conventional clinical prognostic factors, including age, tumor size, tumor grade, ER status, and lymph node status, in addition to GSAS. Tumor grade was converted into a binary factor by assigning grade 2 and grade 3 tumors into one group and grade 1 tumors into the other. Kaplan-Meier curves were generated to visualize the results derived from the Cox proportional hazards models. Specifically, GSASs were dichotomized about 0 reflecting high (>0) and low (<0) gene set activity to stratify patients into two groups and then Kaplan-Meier curves were generated for each patient group.

Survival analyses were implemented in the R programming language using the “survival” package. The “survreg” and “coxph” functions were used to create the Cox proportional hazards models. The “survdiff” function was used to compare the difference between the positive and negative GSAS groups in the Kaplan-Meier curves.

### Random forest model for predicting metastasis

A Random Forest classifier was trained using gene sets that significantly differed in GSAS between metastatic and non-metastatic samples as features (Wilcoxon ranked sum test, FDR < 0.01). Gene set features were selected from one dataset and used to predict metastasis status of samples from a second validation dataset. The performance of the model, using these features, was evaluated in the second dataset using 10-fold cross-validation. Specifically, samples were randomly divided into 10 subsets with 9 subsets being used to train the classifier and predict the metastasis status of the remaining validation set. This process was repeated 10 times so that all samples were in the validation set once. A receiver operating characteristic (ROC) curve was generated and the area under the curve (AUC) was calculated to assess model effectiveness. The overall cross-validation procedure was iterated 10 times and to obtain an average AUC score. Random Forest analyses were performed in R using the “randomForest” package.

### Functional enrichment analysis

Genes from significant signatures were inputted into DAVID (http://david.abcc.ncifcrf.gov) [30] and run against a *Homo sapiens* gene background. Functional annotation clustering was used to group genes with similar functions together and was run at a classification stringency of “medium”.

### Construction of overlapping network for gene sets

Gene overlap was examined in gene sets that were significantly correlated with breast cancer survival in the van de Vijver dataset (FDR < 0.01). Gene sets were separated into two groups based on hazard ratio (hr) in the van de Vijver dataset, with gene sets with a hr ≥ 1.00 constituting a “negative” set and gene sets with a hr < 1.00 constituting a “positive” set. Further analysis was performed separately on each set. An overlap score was calculated by comparing the number of genes shared in common between each gene set and dividing it by the union of the genes contained in the two gene sets. This process was repeated until the overlap score for all possible pairs of signatures in a set had been calculated. Signature pairings with overlap scores less than 0.20 were then filtered out of the data. The resulting datasets were visualized using Cytoscape with each node representing a different signature. Node size was scaled to the p-values of calculated from the survival analysis, with larger nodes corresponding to smaller p-values. Edge length was scaled to the overlap score, with shorter edge lengths indicating higher overlap scores. Significant gene sets across all seven datasets (p ≤ 0.05) were highlighted within the network.

### Network module selection and the core gene set

Modules in the network were identified qualitatively based on node clustering patterns. A single network module rich in gene sets significantly associated with patient survival across all datasets was selected for further analysis. Genes present in at least 40% of the gene sets in the module of interest were selected for. These genes made up the module’s “core gene set”. The GSAS for this core gene set was calculated and subjected to survival analysis as described above. For Random Forest classification, a Wilcoxon ranked sum test was performed to measure the difference between core gene set gene expression levels in the metastatic and non-metastatic group. Genes that significantly differed (FDR < 0.01) between the two groups were selected as features. From there, the Random Forest classification procedure was followed as described above. The resulting core gene set was also examined for functional enrichment using DAVID, as described above.

### Survival analysis after removal of the core gene set

The genes of the core gene set were removed from the gene sets downloaded from MSigDB using two approaches. The first approach is similar to a technique reported by Donato, et al. to analyze crosstalk between pathways [31]. In this approach, the genes of the core gene set were removed from all gene sets without replacement. In the second approach, each gene set from MSigDB that shared genes with the core gene set had the shared genes substituted with random genes that were not already present in the gene set. The GSASs for the resulting gene sets from each method were then recalculated and fitted with a univariate Cox proportional hazards model.

## Results

### Activity scores of MSigDB gene sets in breast cancer patients

**Figure 1:**
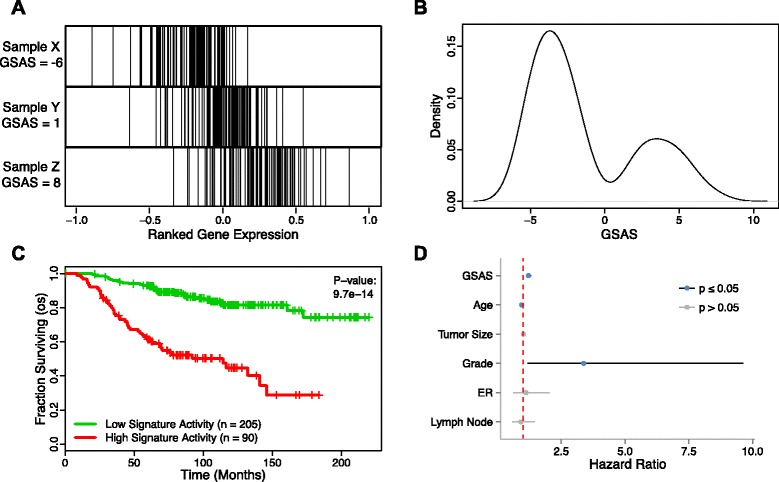
**The GSAS of VANTVEER_BREAST_CANCER_METASTASIS_DN predicts patient survival. (A)** The distribution of genes from this gene set in an expression-ranked gene list in samples with a low (Sample X), intermediate (Sample Y), and high (Sample Z) GSAS. **(B)** The distribution of GSASs across all samples in a dataset. **(C)** Patients with positive GSASs (red curve) show significantly shorter survival times than those with negative GSASs (green curve). Vertical hash marks indicate points of censored data. **(D)** In a Cox PH model, this GSAS significantly predicts patient survival even after adjusting for traditional clinical features. A red dotted line indicates where the hazard ratio = 1.

Using the gene expression data provided by van de Vijver et al. [27], we calculated the gene set activity score (GSAS) for each gene set contained in the C2 curated gene set collection of MSigDB. Briefly, the GSAS for a sample is determined by calculating either the maximum positive or negative deviation between two empirical distribution functions. The foreground function is based on the position of the genes of a gene set in a list of genes rank-ordered by the gene expression values from the sample, while the background function is based on the position of the genes not found in the target gene set. Thus, a negative GSAS indicates low gene set activity, due to low relative expression levels of the component genes, while a positive GSAS indicates high gene set activity, due to high relative expression levels of the component genes. Figure 1A demonstrates the gene distribution of three samples exhibiting either a low (GSAS = −6), intermediate (GSAS = 1) or high (GSAS = 8) GSAS for a well-known breast cancer gene signature reported by van’t Veer et al. [2]. As expected, the genes in the sample with the low score cluster toward the left, while the genes in the sample with the high score cluster toward the right. Gene sets which have a GSAS around zero have component genes whose expression values are evenly distributed about zero. For each gene set this method was applied to, the resulting distribution of GSASs across all samples followed a bimodal distribution. This can be seen in the distribution of GSASs for the signature reported by van’t Veer et al. (Figure 1B).

### Survival analysis using GSAS

The GSAS is useful when performing survival analysis because it can be used to stratify samples based on the activity level of a gene set’s component genes in a given sample. Gene sets in the MSigDB C2 curated gene set collection tended to be representative of biological pathways or molecular signatures associated with a biological function or disease state. As a result, the GSAS provided a glimpse of the underlying biology of each sample. Because the GSASs of the van’t Veer gene set followed a bimodal distribution with a local minimum at approximately 0, samples were dichotomized by GSAS, with one group containing samples with GSASs < 0 and one containing samples with GSAS > 0. For the van’t Veer gene set, samples with a GSAS > 0 demonstrated significantly shorter survival time than samples with a GSAS < 0 (p = 9.7e-14) (Figure 1C). When fitted with a multivariate cox proportional hazards model that took into account other clinical factors including age, grade, tumor size, ER status, and lymph node status, GSAS for this gene set remained significant (p = 1.3e-6) with a hazard ratio of 1.21 (Figure 1D).

**Figure 2:**
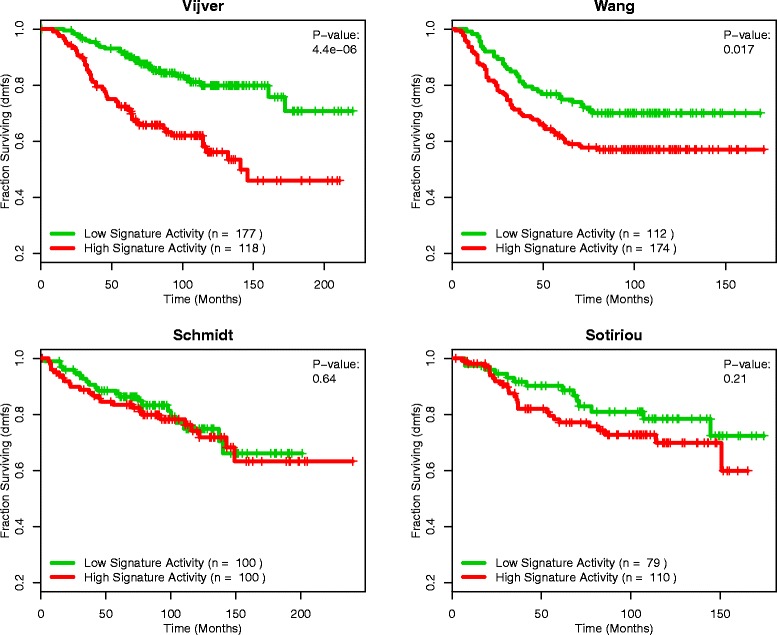
**KARAKAS_TGFB1_SIGNALING as a predictor of survival in four datasets.** Patients with positive GSASs (red curve) versus negative GSASs (green curve) for the gene set KARAKAS_TGFB1_SIGNALING in four different datasets. The GSASs for this gene set are significantly predictive of survival in the van de Vijver and Wang datasets (p < 0.05), however when applied to the Schmidt and Sotiriou datasets, the GSASs for this gene set are no longer significant (p > 0.1).

Several gene sets available on MSigDB have been reported to be significantly associated with breast cancer survival, while others represent pathways that may be significantly associated with patient survival. However, many of these signatures and pathways are not robust in predicting clinical outcome across large groups of patients, and their predictive value can be highly dependent on the dataset from which they are derived [32,33]. Figure 2 shows an example of this, with samples from four publicly available datasets [8,10,27,28] dichotomized based on their GSAS from a gene set containing genes upregulated by transforming growth factor-beta 1, a protein involved in the regulation of cell proliferation [34]. While the gene set activity was significantly correlated with distant metastasis-free survival in the van de Vijver and Wang datasets (p = 4.4e-6 and 0.02, respectively), there appeared to be little separation between samples with high and low gene set activity in the Schmidt and Sotiriou datasets (p > 0.1 for both).

### Identification of robust gene sets

**Figure 3:**
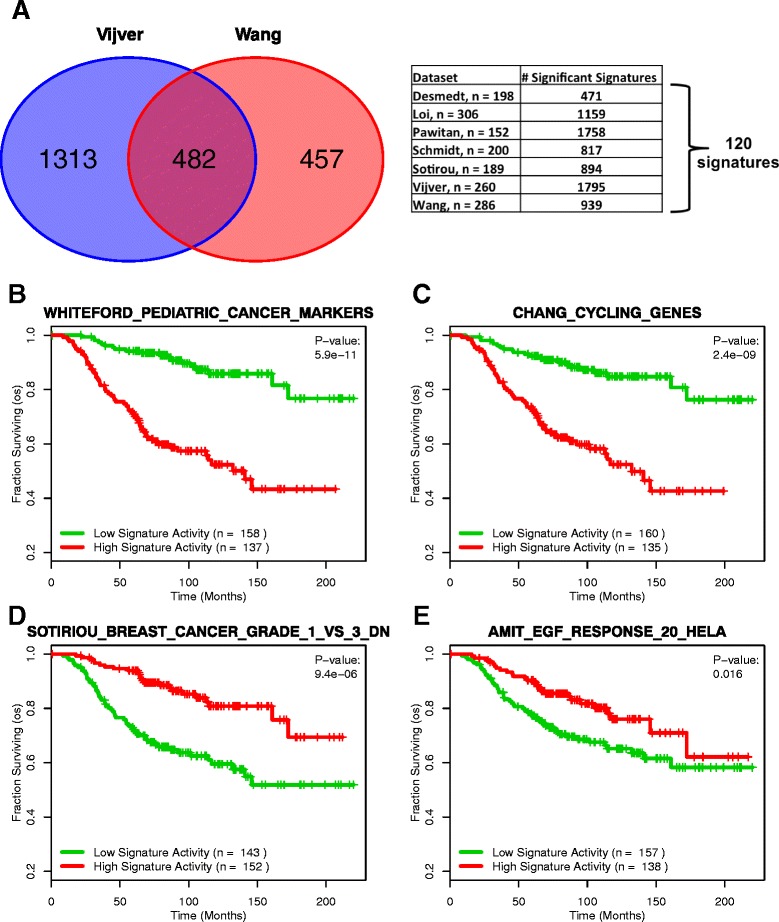
**Expansion of analysis to 7 datasets yields 120 robust signatures. (A)** Overlap analysis of gene sets significantly correlated with survival across seven datasets (p < 0.05) reveals 120 “robust” gene sets. **(B)** A gene signature containing genes that are differentially expressed between pediatric tumors and normal tissue. **(C)** A gene signature whose genes are associated with periodic cell cycle expression. **(D)** A gene signature whose genes are associated with histologic grade. **(E)** A gene signature containing genes stimulated by EGF in HeLa cells. **(B-E)** are examples of the 120 robust gene sets. In **(B)** and **(C)**, high signature activity has a protective effect (hr < 1), while in **(D)** and **(E)** high signature activity has a deleterious effect (hr > 1).

Gene sets whose predictive value is localized to only a few datasets are not of much use clinically, as the association between patient survival and gene set activity may be due to confounding factors in the datasets or a small overall patient sample size. Gene sets with consistent prognostic performance across many datasets are less likely to have these problems, as they have been tested over a more diverse setting with a greater overall patient sample size. To identify gene sets such as these, we first extended our analysis done in the van de Vijver et al. dataset (see above) to four more datasets [8,10,28,29] and two sub-datasets derived from a meta-analysis study [26] for a total of seven independent analyses. Each analysis yielded a list of gene sets ranked by its fit in a Cox proportional hazards model based off the given dataset’s patient survival information. We then identified gene sets that remained predictive of survival across all seven datasets by selecting for the ones that had a Cox proportional hazards p-value < 0.05 in every analysis. This yielded a list of 120 gene sets that were significantly associated with patient survival across every dataset we tested (Figure 3A, Additional file 1). Examples of some of these robustly predictive gene sets are shown in Figures 3B-E as survival curves generated from the van de Vijver dataset. The two gene sets in Figures 3B and C have a hazard ratio greater than 1 (hr = 1.33 and 1.26, respectively). In these gene sets, higher activity is associated with decreased survival, indicating that these gene sets are representative of a deleterious expression signature. The opposite is shown in the gene sets represented in Figures 3D and E, in which both gene sets have a hazard ratio less than 1 (hr = 0.75 and 0.83, respectively). For these gene sets, increased gene set activity is associated with increased survival time. This suggests that these gene sets are indicative of a gene expression profile exhibited by patients having a favorable prognosis.

We followed up this analysis by using DAVID Functional Annotation Clustering [30] to investigate whether the genes present across these gene sets shared similar functions. This would allow us to ascertain whether these gene sets were consistently predictive because they represented a common cancer associated process, or because they encompassed several unique functions. The 120 gene sets first had to be separated based on hazard ratio from the van de Vijver et al dataset into a deleterious group (hr > 1) and a protective group (hr < 1) to eliminate noise that would occur if the two groups were examined together. The genes in these two groups were then compiled into separate lists and examined using DAVID. Due to the large number of genes in the deleterious group, we were required to reduce the size of the gene list. We chose to do so by selecting the genes that showed up in at least 5% of the deleterious gene sets. This procedure was not necessary for the protective group due to its smaller size. Our analysis showed that genes from both the deleterious group and the protective group were enriched in genes whose functions related to cell cycle regulation (Additional files 2 and 3).

**Figure 4:**
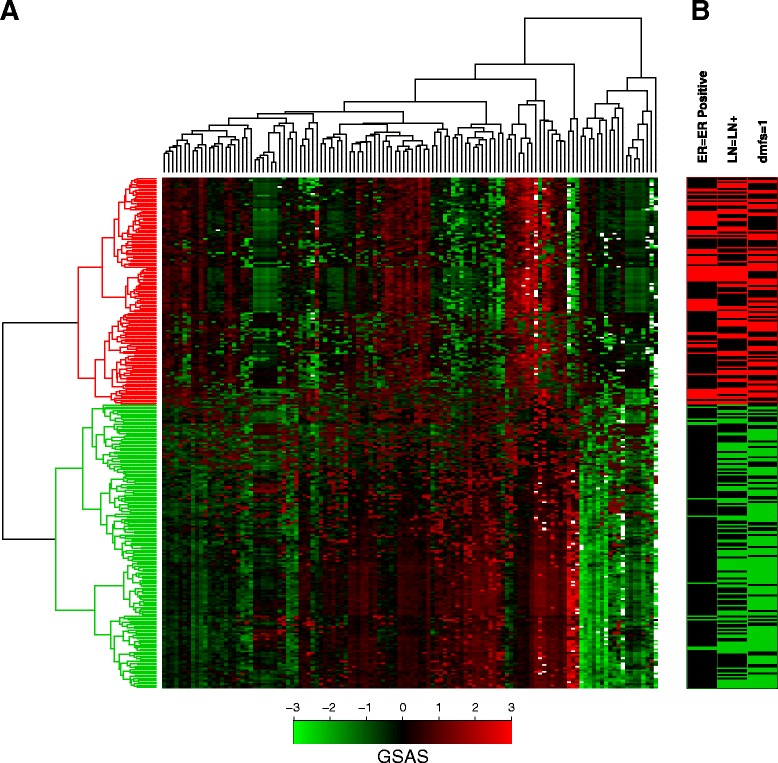
**Hierarchical clustering of samples using the GSASs for the 120 robust gene sets. (A)** A heatmap showing the pattern of GSASs for the 120 robust gene sets across the 295 samples in the van de Vijver dataset. Each row represents one sample’s GSAS profile for each of the 120 robust gene sets and each column represents the GSASs across all samples for one of the robust gene sets. To show contrast, all GSASs less than −3 or greater than 3 were adjusted to −3 and 3, respectively. Green is indicative of a lower (more negative) GSAS for a sample while red is indicative of a higher (more positive) GSAS for a sample. **(B)** Hierarchical clustering of the samples based on GSAS in the robust gene sets reveals two distinct groups of samples. The red group is enriched in samples with ER- breast cancer and distant metastasis occurrence compared to the green group.

We additionally examined whether the GSASs for these gene sets could be used to distinguish clinically relevant subgroups. We hierarchically clustered samples from the van de Vijver et al dataset based on each sample’s GSAS for each of the gene sets tested. We then looked at whether clinical features such as ER status, lymph node metastasis status, and distant metastasis occurrence clustered as well. Figure 4 displays a heatmap detailing the results of this analysis. Samples were split into a red group and a green group based on where they clustered. The red group was enriched in samples with ER- breast cancer and distant metastasis occurrence relative to the green group, both indicators of more severe disease. The trends observed here suggest that GSASs from the robust gene sets are capturing clinically relevant processes in addition to survival information.

### Metastasis classification using metastasis-associated GSASs

**Figure 5:**
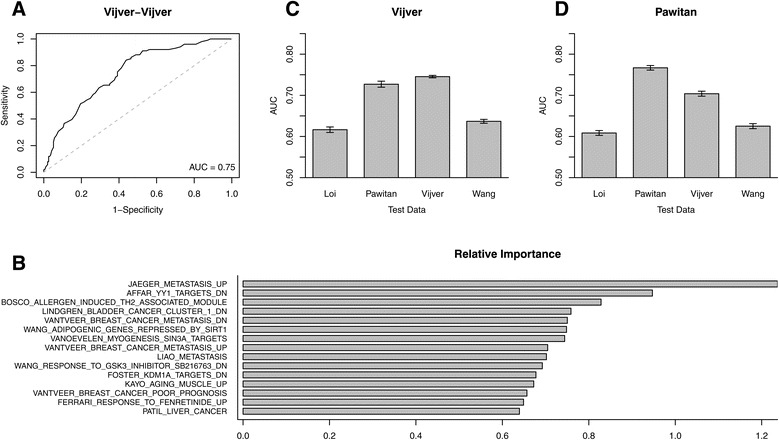
**Metastasis prediction performance using GSAS. (A)** A receiver operating characteristic (ROC) curve for the Random Forest classification of metastatic versus non-metastatic samples in the van de Vijver dataset using GSASs that significantly differed between metastatic and non-metastatic samples in the van de Vijver dataset (Wilcoxon rank-sum test, FDR < 0.01) as the training data. **(B)** The relative importance values assigned by the Random Forest classifier used in **(A)** to each gene set when classifying samples. **(C, D)** AUC scores for the Random Forest classification of metastatic versus non-metastatic samples in different datasets when using GSASs that significantly differed between metastatic and non-metastatic samples in van de Vijver **(C)** and Pawitan **(D)** as the training data.

We next tested how informative GSASs were in metastasis classification across datasets using a Random Forest classifier. We selected gene sets that significantly differed between metastatic and non-metastatic samples as features since the full array of 4,257 gene sets contained many gene sets unrelated to metastasis and cancer (see Methods). We began this analysis in the van de Vijver dataset, where 520 gene sets qualified to be used as features. 10-fold cross validation was then performed to assess the classifier’s accuracy at predicting metastasis status for samples from the same dataset. The area under the ROC curve (AUC) was chosen to measure classifier performance in terms of specificity and sensitivity values. Our analysis on the van de Vijver dataset yielded an AUC of 0.75, which suggests relatively good performance (Figure 5A). The relative importance assigned to each gene set by the Random Forest classifier can be seen in Figure 5B.

To demonstrate the reproducibility of this classification method we expanded our analysis to three additional datasets, using the 520 gene sets identified from the van de Vijver dataset as features to predict metastasis status in samples from the Loi, Pawitan, and Wang datasets. The resulting analyses yielded AUCs of 0.62, 0.72, and 0.64, respectively, which indicate relatively accurate cross-dataset prediction (Figure 5C). To show that these results were not dependent on features selected from the van de Vijver dataset we repeated this analysis, this time selecting gene sets from the Pawitan dataset. The resulting 790 gene sets that qualified were used as features to classify metastasis status in samples from the Loi, Pawitan, van de Vijver, and Wang datasets. This resulted in AUCs of 0.61, 0.77, 0.70, and 0.63, respectively, again suggesting relatively high performance (Figure 5D). Taken together, our AUCs were very similar to those achieved in a study done by Chuang et al that used protein-protein interaction subnetwork activity scores from the van de Vijver dataset to predict metastasis status in the Wang dataset, and vice versa [35]. The high performance achieved using GSAS-based classifier features suggests that the GSAS is successfully capturing information about the behavior of the component genes in a given gene set, allowing GSAS to serve as an unbiased marker of metastasis risk.

### Gene set network analysis and creation of the core gene set

**Figure 6:**
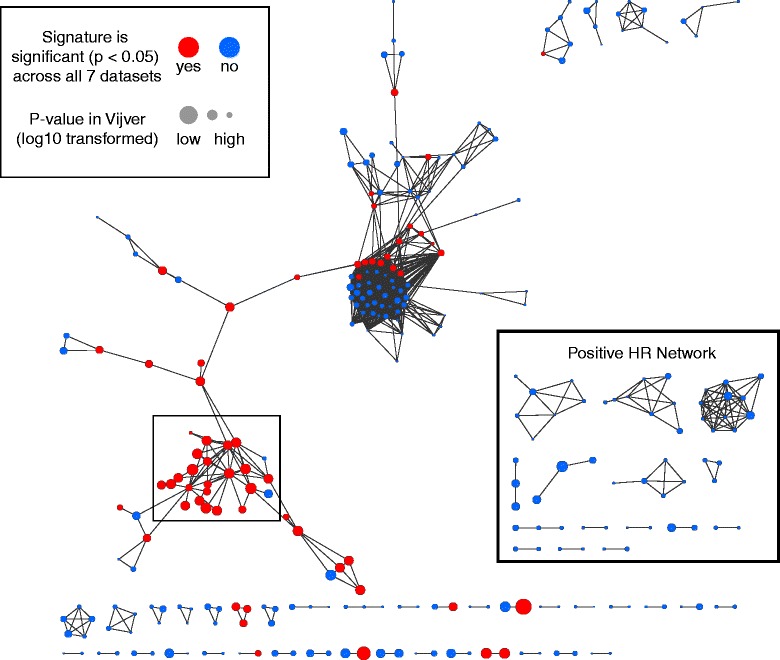
**A gene set network reveals a module highly enriched in cell proliferation genes.** Gene sets significantly associated with survival (FDR < 0.01) in the van de Vijver dataset were selected and dichotomized based on having a negative effect hazard ratio (hr ≥ 1.00) or a positive effect hazard ratio (hr < 1.00). A network was then created from the two groups of genes linking the gene sets (nodes) by the number of genes they had in common (edges). This analysis revealed a module (solid box) made up of the robust gene sets whose genes were enriched in cell proliferative-based functions.

Our success in using GSASs to characterize the gene sets of MSigDB led us to seek ways to streamline our analyses further. We reasoned that the GSASs from a gene set may be correlated with patient survival due to a few strong survival-associated genes rather than the overall behavior of the gene set. To test this, we investigated the shared-gene relationship among the gene sets, as genetic similarity between gene sets may imply comparable prognostic association. We applied a network-based approach (see Methods), to visualize this relationship between survival-associated gene sets, which would allow us to better identify collections of gene sets that have many genes in common (Figure 6). Briefly, each node in the network represented a gene set that was associated with survival in the van de Vijver dataset (FDR < 0.01), with an edge present between gene sets if the number of genes the two gene sets shared divided by the number of genes in the union of the gene sets was > 0.20. We identified a module in the network that was enriched in the robust gene sets we had described earlier (solid box, Figure 6), which indicated that many of these gene sets have high gene overlap. To characterize the prognostic contribution of these shared genes we selected for the genes that were present in at least 40% of the module gene sets to create what we termed the module’s core gene set (Additional file 4).

To understand the biological context of the core gene set, the gene set’s component genes were inputted into DAVID Functional Annotation Clustering [30]. The genes of the core gene set were grouped into clusters relating to mitotic cell division, microtubule organization, chromosomal organization, and DNA replication (Additional file 5). These cell proliferation-based functions all play a strong role in cancer progression, indicating that this gene set may be highly associated with patient survival.

### Survival analysis of the core gene set

**Figure 7:**
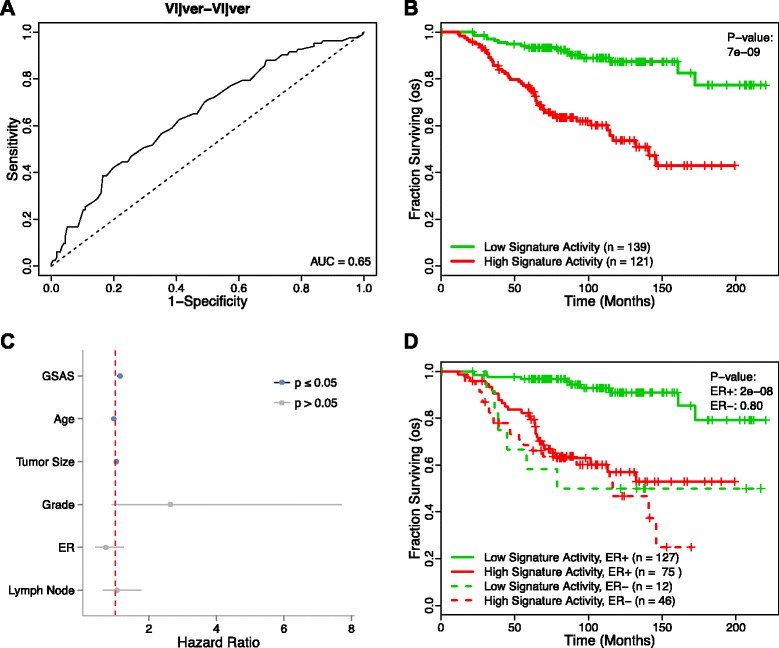
**Performance of the core gene set in classification and survival analysis. (A)** A receiver operating characteristic (ROC) curve for the Random Forest classification of metastatic versus non-metastatic samples in the van de Vijver dataset using the genes of the core gene set whose expression significantly differed between metastatic and non-metastatic samples in the van de Vijver dataset (Wilcoxon rank-sum, FDR < 0.01) as the features. **(B)** Patients from the van de Vijver dataset with a positive GSAS for the core gene set (red curve) show significantly shorter survival times than those with a negative GSAS (green curve). Vertical hash marks indicate points of censored data. **(C)** In a Cox PH model, the GSAS for the core gene set significantly predicts patient survival even after adjusting for traditional clinical features. **(D)** The core gene set is effective in predicting patient survival in ER+ samples but not in ER- samples.

To characterize the role of the core gene set in breast cancer prognosis, we first evaluated the gene set’s ability to classify samples in the van de Vijver dataset as metastatic or non-metastatic. We repeated our Random Forest analysis as described previously, but instead used the core gene set’s component gene expression values as features. Our analysis achieved an AUC score of 0.65 (Figure 7A), a relatively good score for a single gene set, which suggests that the core gene set is ably capturing metastasis information.

We followed up this analysis by using BASE to calculate the GSAS for this gene set in patients from the van de Vijver dataset. A Cox proportional hazards model was used to correlate the core gene set’s activity with patient survival time, and a Kaplan-Meier plot was developed to depict this analysis by stratifying patients by GSAS about 0 (Figure 7B). This analysis found that the activity of the core gene set was significantly associated with patient survival (p = 7e-9). Furthermore, when the analysis was repeated using a multivariate Cox proportional hazards model that takes into account traditional clinical variables, including age, tumor size, grade, ER status, and lymph node metastasis status, the core gene set GSAS remained significantly predictive (p = 1.6e-4, hr = 1.15) (Figure 7C).

ER status is a traditional marker used in the clinic when determining breast cancer patient prognosis. The high association between the core gene set’s GSAS and patient survival led us to examine whether it could remain predictive across ER+ and ER- subtypes. The results of the comparison between ER status and patient survival are displayed in Figure 7D. This analysis showed that the core gene set was significantly associated with patient survival in patients who were ER+ (p = 2e-8), but no significant correlation was found in patients who were ER- (p > 0.10). This finding was in line with a previous study by Iwamoto et al. that found that there were few robust, prognostic signatures that exist for ER- breast cancers [19].

**Figure 8:**
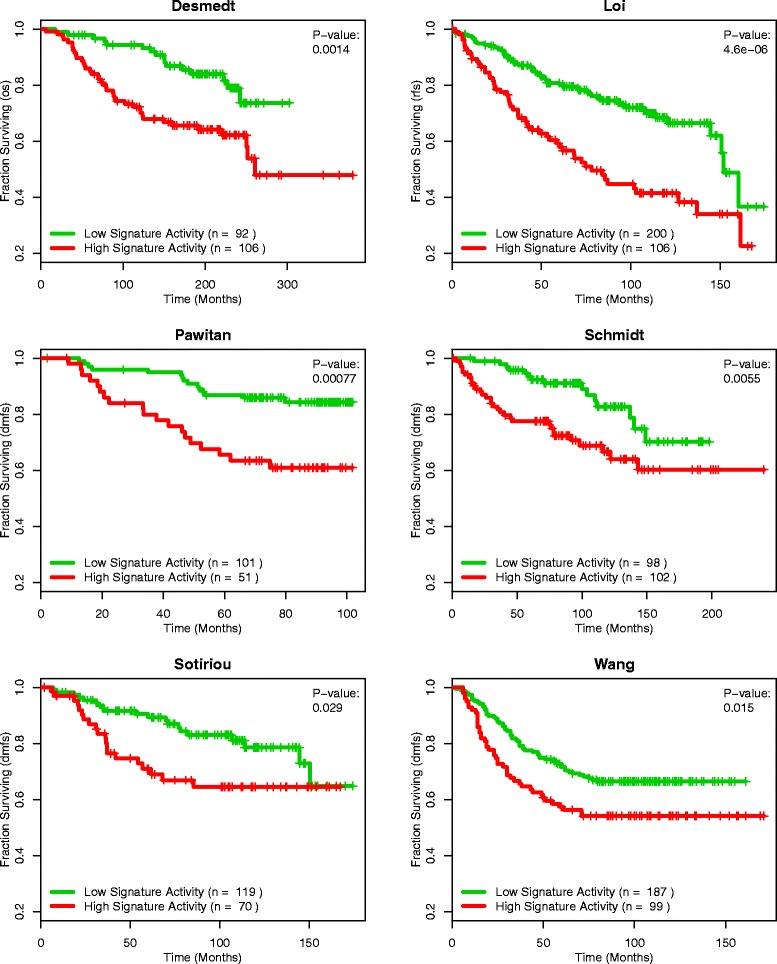
**Survival analysis of the core gene set across datasets.** Across all datasets, patients with positive core gene set GSASs (red curve) show shorter survival times than those with negative core gene set GSASs (green curve) (all p-values <0.05). Vertical hash marks indicate points of censored data.

To show that the high prognostic association of the core gene set was not localized to the van de Vijver samples, we expanded our analysis to the remaining datasets. Figure 8 displays the Kaplan-Meier curves generated when stratifying the samples of each dataset by their core gene set GSAS. As with the van de Vijver dataset, the core gene set GSAS was significantly correlated with breast cancer survival outcome (all p-values < 0.05). This analysis indicated that the core gene set was capturing important survival-associated processes that could be used to inform patient prognosis independent of the cohort from which it came.

### Removal of the core gene set genes from other gene sets disrupts their predictive ability

The strong performance of the core gene set in predicting patient survival time furthered our reasoning that the component genes of this gene set were the source of predictive value in other gene sets on MSigDB. In this scenario, a gene set’s predictive ability would be dependent not on the overall behavior of the gene set, but instead on the number of genes from the core gene set it contains. We used two approaches to test this hypothesis. In the first one, genes of the core gene set were removed from all gene sets downloaded from MSigDB. Their GSASs were recalculated and correlated with survival using a Cox proportional hazards model. In the second approach, genes of the core gene set were substituted with random genes so that the number of genes in each gene set from MSigDB remained the same (see Methods). The GSASs were then recalculated and correlated with patient survival as described in the first method.

The first approach resulted in a reduction in the number of gene sets significantly associated with survival (p < 0.05) across all datasets. For the Desmedt, Sotiriou, and Schmidt datasets, the removal of the core gene set genes resulted in no gene sets being significantly associated with breast cancer survival. For the Loi, Pawitan, van de Vijver, and Wang datasets, the number of significant gene sets was reduced (n = 1159 to n = 214 in Loi, n = 1758 to n = 1087 in Pawitan, n = 1795 to n = 1176 in van de Vijver, and n = 939 to n = 165 in Wang). The results from the second approach were slightly more dramatic. When the core gene set genes in each gene set were substituted with random genes, the Desmedt, Sotiriou, and Schmidt datasets again had no gene sets that were significantly associated with breast cancer survival while the Loi, Pawitan, van de Vijver, and Wang datasets saw a reduction in the number of significant gene sets (n = 1159 to n = 175 in Loi, n = 1758 to n = 1068 in Pawitan, n = 1795 to n = 1164 in van de Vijver, and n = 939 to n = 149 in Wang) (Table 1). Taken together, these results suggest that many of the gene sets on MSigDB derive their prognostic value from the component genes of the core gene set.

**Table 1:** **Survival-associated gene sets before and after removal of the core gene set**

	**p < 0.05**	**Removed**	**Substituted**
Desmedt	471	0	0
Loi	1159	214	175
Pawitan	1758	1087	1068
Schmidt	817	0	0
Sotiriou	894	0	0
van de Vijver	1795	1176	1164
Wang	939	165	149

For all datasets, the number of survival-associated gene sets (p < 0.05, Cox proportional hazards model) before (Column 1) and after removal of the core gene set (Column 2) or substitution of the genes of the core gene set with random genes (Column 3).

## Discussion

Currently, the use of predefined gene signatures for informing breast cancer prognosis is dependent on the absolute expression levels of the signature’s component genes in a patient. The assumption is that the signature’s component gene expression levels, either individually or combined, can provide meaningful information about the patient’s expected survival time. However, due to the genetic heterogeneity of cancer, this assumption may not always hold true, resulting in decreased prognostic accuracy. Additionally, many predefined gene signatures have been developed by selecting for genes whose expression values are statistically correlated with cancer survival in a single dataset. The relatively small sample size of patients used in these experiments may provide an inaccurate representation of cancer at a global level, leading to signatures that are predictive in one dataset, but not predictive when applied to others.

The scoring system we have proposed here measures the activity of a gene set based on the component genes’ relative position in a list of genes ranked by a patient’s expression levels. This method is beneficial for numerous reasons. First, it provides a medium to compare gene set activity levels for samples across datasets. In the context of survival analyses, the system allows for the identification of robust gene sets based on the association of a gene set’s GSASs with survival across multiple datasets. Additionally, the score can account for different subsets of a gene set being active. For example, in a pathway-based gene set, one sample that has a slight cumulative increase in the overall expression levels of the pathway genes could potentially have the same score as a sample with a sharp increase in the expression levels of only the downstream genes. In this way, the GSAS mitigates the overfitting problem associated with comparing individual gene set component gene expression levels. Furthermore, the activity score accounts for the effect of gene-gene interactions and potential redundant expression changes. Tumorigenesis typically begins with clonal expansion of cells that have gained a set of driver mutations. As the regulatory mechanisms in the cells are disrupted, tumor cells can pick up passenger mutations that may obscure the causative mutations in a gene expression study. The GSAS can illuminate which mechanisms are important in driving cancer growth by measuring expression change at a functional gene set level, as opposed to an individual gene level, where gene-gene interactions complicate the information that can be derived based on expression data. This feature manifests itself in our classification of non-metastatic versus metastatic samples. By using gene sets with differential activity scores between non-metastatic and metastatic samples to predict metastasis status, we achieve clinically relevant accuracy scores that are reproducible across datasets.

The gene set overlap network analysis we performed allowed for the identification of a novel gene set, referred to as the core gene set, that was enriched in proliferative functions. This gene set was made up of genes that were shared across many robustly predictive gene sets. Survival analysis using the GSAS for this gene set found that the core gene set was significantly associated with survival with a hazard ratio > 1 in all seven datasets in which it was tested. These findings were in agreement with a previous study that claimed that increased proliferative potential of a cell could result in more severe stages of cancer [22]. After removing the core gene set’s component genes from the gene sets on MSigDB we found that many of the survival-associated gene sets were no longer predictive. This suggests that a gene set’s prognostic ability is instead based on the number of core genes it contains. This is of great interest, as it is contrary to the common belief that gene sets derive their predictive ability from the process or pathway they represent. Based on our findings, the core gene set may represent a series of cancer driver genes that directly impact a patient’s cancer severity. In this scenario, the core gene set could also represent a list of potential drug targets. Because high core gene set activity is associated with worse prognosis, drugs that reduce the expression levels of these genes to near normal levels could have the potential to improve patient survival time.

Breast cancer is a molecularly heterogeneous disease, with no two cases of breast cancer being quite the same. Molecular subtypes of breast cancer have been identified based on the genetic profiles of many different patients to help elucidate this heterogeneity [36]. While our GSAS analyses did include multivariate Cox proportional hazards models that account for estrogen receptor status and lymph node metastasis in the van de Vijver dataset, we did not stratify patients by molecular subtype of breast cancer when comparing good and poor prognosis groups. Stratifying samples by molecular subtype before performing survival analyses may increase the resolution between good and poor prognosis groups, yielding new gene sets that are predictive of breast cancer survival in a specific subtype. These analyses could result in a unique set of significant signatures for each subtype, as well as a unique core gene set enriched for functions specific to that subtype.

Going forward, we believe the GSAS survival analysis we have detailed could have applications to cancers outside of breast cancer. For example, this analysis could be used to reveal the prognostic gene sets in other cancers. Identifying survival-associated gene sets in a pan-cancer manner could shed light on the links between cancers while also highlighting differences in their molecular composition. Additionally, the GSAS survival analysis could be used to apply the core gene set detailed in this paper to patient cohorts suffering from other cancer types. This analysis would further characterize the core gene set as a list of cancer driver genes, and would allow us to assess whether its prognostic significance is universal or restricted only to breast cancer.

## Conclusion

This paper presents a powerful method to correlate gene set activity with patient survival. Because the GSAS scoring system is based on relative gene expression as opposed to absolute gene expression, it can be used to compare survival significance across datasets, identify robustly prognostic gene sets, and predict metastasis status in samples in an accurate and reproducible manner. Additionally, the core gene set analysis detailed in this paper indicates that only a small collection of genes drive survival prediction in breast cancer. This suggests that the predictive ability of a given gene set is strongly dependent on the number of core gene set genes present in the gene set. As more gene sets are discovered and tools for measuring gene expression improve, we are hopeful that the association between patient survival and gene set activity level will become even more distinct, allowing for improved prognostic prediction and clinical characterization.

## Additional files

Additional file 1:
**List of gene sets significantly correlated with breast cancer survival across all seven datasets.**
Additional file 2:
**DAVID Functional Annotation Clustering analysis of deleterious genes from the robust gene sets.**
Additional file 3:
**DAVID Functional Annotation Clustering analysis of protective genes from the robust gene sets.**
Additional file 4:
**List of the genes in the core gene set.**
Additional file 5:
**DAVID Functional Annotation Clustering analysis of the genes of the core gene set.**
